# How Health Policies Shape Antibiotic Use: A Nationwide Study of “Access”-Group Antibiotics Availability and Consumption in a Middle-Income Country: The Case of Kazakhstan

**DOI:** 10.3390/antibiotics15060585

**Published:** 2026-06-08

**Authors:** Antonio Sarria-Santamera, Márió Gajdács, Aliya Raimbayeva, Tatyana Belikhina, Lyudmila Pivina, Nurlan Aukenov, Larissa Makalkina, Nurgul Aldiyarova, Yuliya Semenova

**Affiliations:** 1 School of Medicine, Nazarbayev University, Astana 010000, Kazakhstan; antonio.sarria@nu.edu.kz (A.S.-S.); aliya.raimbayeva@nu.edu.kz (A.R.); yuliya.semenova@nu.edu.kz (Y.S.); 2 Department of Public Health, Albert Szent-Györgyi Medical School, University of Szeged, 6725 Szeged, Hungary; gajdacs.mario@med.u-szeged.hu; 3 MTA-SZTE Lendület “Momentum” Anthropogenic Stress and Plant Resilience Research Group, Közép Fasor 52, 6726 Szeged, Hungary; 4 Department of Science, Medical Center Hospital of the President’s Affairs Administration of the Republic of Kazakhstan, Astana 010000, Kazakhstan; 5 Department of Emergency Medicine, Semey Medical University, Semey 071407, Kazakhstan; lyudmila.pivina@smu.edu.kz; 6 Research Administration, South Kazakhstan Medical Academy, Shymkent 160001, Kazakhstan; n.aukenov@skma.kz; 7 Department of Clinical Pharmacology, Astana Medical University, Astana 010000, Kazakhstan; 8 Professional Association of Clinical Pharmacologists and Pharmacists, Astana 010000, Kazakhstan; nurgul.aldiyarova@gmail.com

**Keywords:** antibiotic consumption, antimicrobial stewardship, AWaRe classification, defined daily doses, DDD, drug registration, primary care, Kazakhstan

## Abstract

**Background/Objectives**: Few studies have examined how health policies may influence antibiotic consumption at a global level, particularly with respect to the World Health Organization (WHO) “Access” group antibiotics. Our present study aims to address this gap by investigating how health policies affect the availability of “Access” antibiotics in a middle-income country of Kazakhstan. **Methods**: This study employed a mixed-methods approach: the quantitative component consisted of a time series analysis of “Access” antibiotic consumption within the period 2017–2024; the qualitative component involved an analysis of health policies centered on regulating the availability of “Access” antibiotics and their prescription at the primary care level. **Results**: A declining trend in the consumption of “Access” antibiotics was observed during the period from 2017 to 2023; however, in 2024 there was a rebound in consumption. The consumption of “Access” antibiotics did not reach the 60% threshold recommended by the WHO. A total of 50 “Access” antibiotics were found to be unavailable in the country, of which 9 are included in the WHO Essential Medicines List. Analysis of health policies highlighted that the registration of medicines may only be initiated by the manufacturer or its representative. Furthermore, analysis of national standards of care for common infections in primary care indicated a preference for prescribing “Watch” antibiotics, or prescribing antibiotics where their use would not be indicated. **Conclusions**: A coordinated policy response is needed in Kazakhstan to strengthen antimicrobial stewardship, including improving the availability of “Access” antibiotics, decentralization, revising national standards of care, and addressing regulatory barriers to medicine registration.

## 1. Introduction

Antimicrobials are indispensable drugs, allowing humanity to treat previously debilitating or fatal infections, and have allowed for the emergence of various medical fields, such as invasive surgery, intensive care medicine, and neonatology [1]. Antimicrobial resistance (AMR) is a growing public health problem, threatening the sustainable functioning of our healthcare systems, with projections from the Global Burden of Disease (GBD) Study suggesting that bacterial AMR may be responsible for around 1.9 million deaths annually, directly attributable to resistant infections, and up to 8.2 million deaths associated with resistant infections worldwide by 2050, if current trends persist [2].

The non-prudent use of antimicrobials has been described as one of the key drivers of the global AMR crisis [3]. Based on the findings of the World Health Organization’s (WHO) most recent Global Antimicrobial Resistance and Use Surveillance System (GLASS) report, 1 in 6 laboratory-confirmed bacterial infections were antibiotic-resistant [4]. Given that the current antimicrobial research and development (R&D) pipeline remains insufficient to meet future needs, preserving the effectiveness of existing treatments has become a critical public health and clinical priority [5]. To this end, antimicrobial stewardship (AMS) initiatives have emerged as a practical and necessary approach to promote more responsible use of antimicrobials, improve patient outcomes, and reduce the spread of AMR across healthcare settings [6].

The severity of AMR-related issues has been acknowledged by political interest-holders globally: the United Nations General Assembly (UNGA) in 2024 has adopted a political declaration against AMR, where commitments were made by Member State representatives to reduce mortality associated with bacterial AMR by 10%, to reduce inappropriate antimicrobial use in the human health sector by 20% by 2030 (vs. the 2019 baseline), and to ensure 60% of countries have functioning national action plans (NAPs) for AMR [7]. One of the key elements of AMS initiatives is the approach proposed by the WHO to classify antibiotics into three distinct categories, i.e., to “Access”, “Watch”, and “Reserve” groups (hence, the “AWaRe” classification), based on their potential to drive the emergence of AMR [8]. “Access” group antibiotics have a lower potential to select for resistance, and are generally recommended as first- or second-line treatments for common infections. On the other hand, “Watch” group antibiotics have a higher potential to promote AMR, while antibiotics categorized in the “Reserve” group are intended for use as last-resort options [9]. To guide national AMS programs, the WHO has set specific targets for the proportion of “Access” antibiotics to be used, in proportion to all antibiotics used, with the current goal being at least 60% of total antibiotic consumption [8]. More recently, in line with the UNGA political declaration, the WHO has updated this target, recommending that by 2030, up to 70% of consumed antibiotics nationally should belong to the “Access” group [10].

Therapeutic decisions made by antibiotic prescribers are recognized as a major driver of antibiotic consumption [11]. This is supported by observations that variations in antibiotic use across regions, countries, and healthcare facilities correlate more strongly with prescriber practices than with patient demographics [12,13]. Other factors that may influence antibiotic consumption rates include patient demands [14], cultural and social norms (i.e., normative beliefs) surrounding antibiotic use [15], the status and organization of the healthcare system (including the availability of appropriately trained healthcare staff), and the local availability of antibiotics and diagnostic testing facilities [16]. Healthcare system policies directly affect all of above determinants by shaping prescribing guidelines, regulating licensing and availability of antibiotics, and determining access to and/or financing allocated for diagnostic testing [17].

Despite their importance, few studies have examined how health policies influence antibiotic consumption at a global level, particularly with respect to “Access”-group antibiotics. Such evidence would be crucial in low- and middle-income countries (LMICs), where AMS programs are often in their early stages of development [18]. Therefore, the present study aims to address this gap by investigating how health policy influences the availability of “Access” antibiotics in Kazakhstan, an upper-middle-income country in Central Asia with a centrally regulated healthcare system [19]. Specifically, the study aims to: (i) analyze national trends in the consumption of “Access” antibiotics during 2017–2024; (ii) examine the inclusion of these antibiotics in the national Essential Medicines List (EML); and (iii) assess relevant legislative acts and National Standards of Care (NSoCs) to explore how regulatory and clinical guidance may shape the availability and use of these antibiotics.

## 2. Results

### 2.1. Consumption of Access Group Antibiotics in Kazakhstan

**Figure 1:**
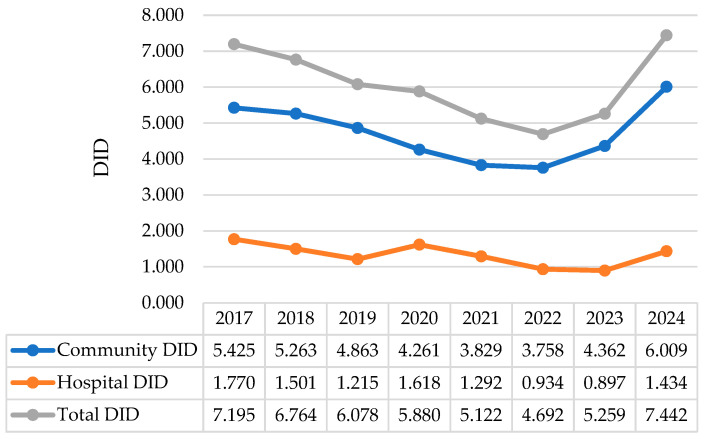
Total, community, and hospital annual “Access”-group antibiotic consumption, expressed as defined daily doses per 1000 inhabitants per day (DIDs).

Figure 1 summarizes the total consumption of “Access” group antibiotics, as well as consumption in the community and hospital sectors in Kazakhstan during 2017–2024, expressed as defined daily doses per 1000 inhabitants per day (DIDs) according to the WHO Global Antimicrobial Resistance and Use Surveillance System (GLASS) framework. In this study, the GLASS sector definitions were applied, where the community sector refers to outpatient and retail pharmacy settings, while the hospital sector refers to inpatient healthcare facilities. Tables S1–S3 showcase consumption categories and trends disaggregated by individual substances.

Over the entire study period (2017–2024), trends in community, hospital, and total consumption were negative, with annual percentage changes (APCs) of −1.30% (95% confidence interval [CI]: −7.79 to 5.63; Mann–Kendall (MK) trend analysis: non-significant trend (*p* = 0.199)) for community consumption, −5.85% (95% CI: −13.13 to 2.04; MK trend analysis: significant decreasing trend (*p* = 0.048)) for hospital consumption, and −2.28% (95% CI: −8.38 to 4.23; MK trend analysis: non-significant trend (*p* = 0.138)) for total consumption, respectively. A recent increase in the consumption of “Access” group antibiotics observed from 2022 onwards may represent a positive development, if accompanied by a proportional reduction in the consumption of “Watch” and “Reserve” group antibiotics. Therefore, monitoring “Access” group antibiotic consumption in relation to non-“Access” antibiotics is crucial to fully appreciate changes in consumption rates in a broader context.

**Figure 2:**
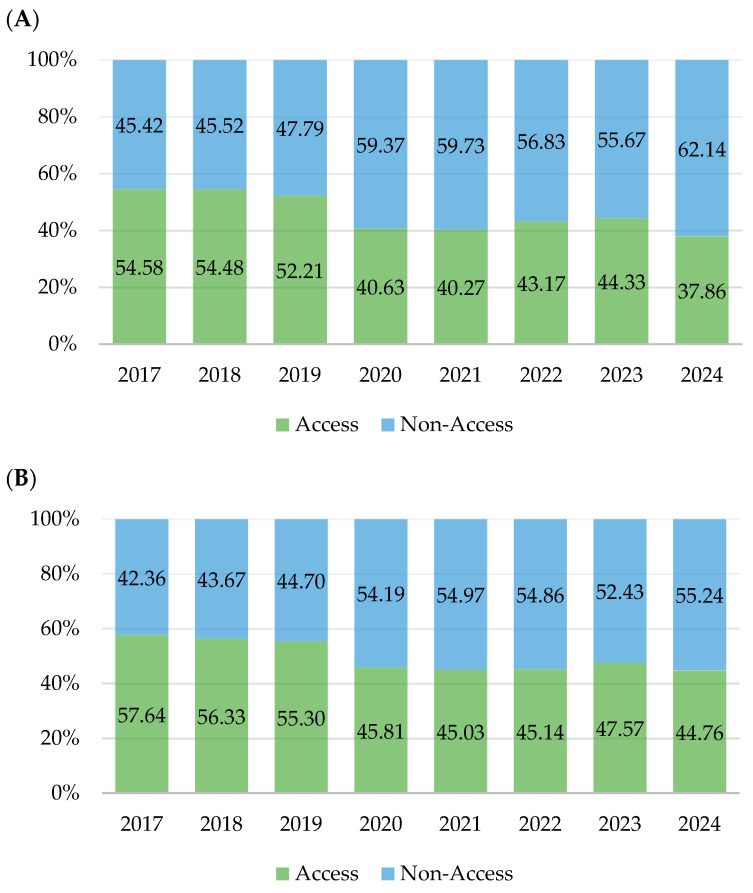

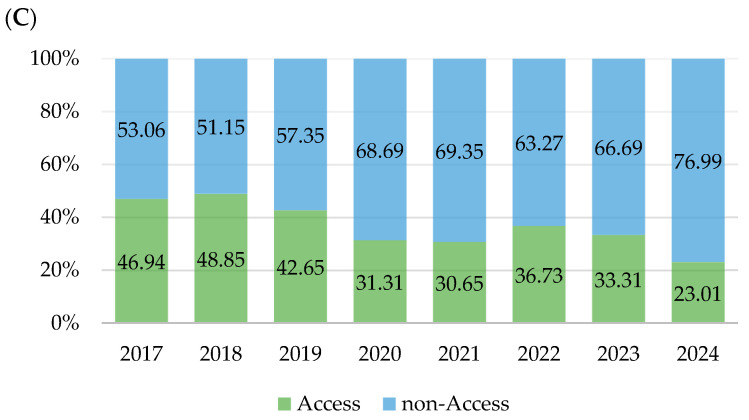
Trends in the consumption of Access group antibiotics relative to non-Access antibiotics in the total (**A**), community (**B**), and hospital (**C**) healthcare sectors.

Figure 2 illustrates trends in the consumption of “Access” group antibiotics during the study period (2017–2024), in relation to the consumption of non-“Access” group antibiotics. Overall, “Access” antibiotics accounted for a relatively smaller proportion of total antibiotic consumption, decreasing from 54.58% to 37.86% over the study period. A similar pattern was observed in both the community sector (57.64–44.76%) and the hospital sector (48.85–23.01%). The MK trend analysis demonstrated a declining trend in the proportion of “Access” antibiotics overall, with an APC of −4.84% (95% CI: −7.93 to −1.65, *p* = 0.005). Similar declines were observed in the community sector (APC: −3.79%, 95% CI: −6.09 to −1.42, *p* = 0.004), while the decrease was even more pronounced in the hospital sector (APC = −8.41%; 95% CI: −13.17 to −3.38, *p* = 0.003).

These declining trends suggest that the increase in overall antibiotic consumption observed during the past two years, expressed in DID, reflects a general rise in antibiotic use across all categories, rather than an increasing share of “Access” group antibiotics.

### 2.2. Availability of “Access”-Group Antibiotics in Kazakhstan and Regulatory Frameworks for Their Registration

In Kazakhstan, the national drug formulary serves as the principal national list of medicines and performs functions of an Essential Medicines List. It is approved by the Ministry of Health (MoH) order and is updated at least once a year; the latest version was approved in November 2025 [20]. The WHO Model List of Essential Medicines (EML) and the WHO Model List of Essential Medicines for Children (EMLc) serve as global reference lists intended to guide countries in the selection of priority medicines for their national health systems. The latest version of EML/EMLc was updated in September 2025 [21].

Nine systemic antibiotics included in the EML/EMLc are not listed in the Kazakhstani national drug formulary. Analysis of antibiotic consumption at the Anatomical Therapeutic Chemical (ATC) classification level 5 indicates that these antibiotics are not marketed in Kazakhstan. Most of these antibiotics belong to the penicillin class, with beta-lactamase-resistant penicillins (ATC code J01CF) being the most underrepresented pharmacological subgroup. Although the fixed-dose combination of sulfamethoxazole and trimethoprim is available, and is widely used in Kazakhstan, neither trimethoprim nor sulfamethoxazole is registered or marketed as a single-agent formulation (Table 1). Supplementary Table S4 summarizes a list of 50 “Access” group systemic antibiotics that are not included in the EML/EMLc and thus are not available in the country.

The limited availability of many “Access” antibiotics in the country raises questions about how the drug registration process is implemented. Table 2 outlines the key provisions of the MoH Order governing drug registration [22]. Only manufacturers, or their authorized representatives, are eligible to initiate the registration process, while government bodies and healthcare facilities are not permitted to do so. In addition, there is no provision for fee waivers, although the registration fees themselves appear relatively modest, amounting to 47,575 national currency units (approximately 95.15 US dollars) for initial registration and 21,625 national currency units (approximately 43.25 US dollars) for re-registration, respectively [23].

### 2.3. Key Provisions of National Standards of Care for Common Infections in Primary Care

Another way to investigate the impact of health policies on the availability of “Access” antibiotics is to examine NSoCs, which are centrally regulated in Kazakhstan, guiding physicians’ clinical decision-making, and underpinning reimbursement mechanisms linked to healthcare service provision [24]. Table 3 presents a summary of key provisions of the NSoC for patients with common infections at the primary care level, in terms of antibiotic prescribing [25], and compares them with the WHO AWaRe Antibiotic Book [26]. Supplementary Table S5 presents detailed information on the indications for antibiotic therapy initiation, overall therapy duration, and recommended doses.

Overall, 20 antibiotics are recommended for the treatment of common infections at the primary care level in the Kazakhstani NSoC, of which 9 (45.0%) belong to the “Access” group, while 11 (55.0%) to the “Watch” group. In comparison, the WHO AWaRe Antibiotic Book recommends 13 antibiotics for the same infections, of which 10 (76.9%) belong to the “Access” group and 3 (23.1%) to the “Watch” group, respectively.

In addition, it was found that the Kazakhstani NSoCs of care recommend antibiotic prescribing for several conditions for which the WHO AWaRe Antibiotic Book either discourages their use or restricts it by specifying clinical criteria. For example, for conditions such as acute sinusitis, chronic bronchitis/exacerbation of chronic obstructive pulmonary disease (COPD), otitis media, pharyngitis, ulcerative gingivitis, and periodontitis, the AWaRe Antibiotic Book indicates that antibiotic treatment is not required in most cases and should only be considered in the presence of complications, severe symptoms, or other clinical indications listed in Table S5.

Three of the antibiotics recommended in the WHO book (phenoxymethylpenicillin, trimethoprim, and cloxacillin) for patients with common infections at the primary care level are not available in Kazakhstan (Table 4). Of interest is the fact that one of the “Access” antibiotics recommended by the NSoC for the treatment of skin abscesses, boils, and carbuncles (oxacillin) is also not available in the country.

## 3. Discussion

Our study has identified pertinent findings related to antibiotic use in Kazakhstan, as well as how policy may influence multiple stakeholders, including national regulatory authorities, manufacturers, and prescribing practices related to antibiotic selection and availability in the country. The most relevant finding involved a decline in the use of certain traditional “Access” antibiotics and a substantial increase in the consumption of specific combination therapies. “Access”-group antibiotic consumption declined over the study period and did not reach the 60% threshold recommended by the WHO [8]. Despite the 2024 increase in “Access” antibiotic volume, their overall share of total consumption declined from 54.58% in 2017 to 37.86% in 2024, indicating that the use of non-“Access” antibiotics grew even more rapidly. Beyond individual substances, the sources highlight that “Watch” antibiotics like azithromycin, ciprofloxacin, and ceftriaxone remained dominant, accounting for nearly 40% of all antibiotic consumption.

This analysis has also identified several essential antibiotics that remain unregistered or unavailable within the Kazakhstani market despite their global importance. Furthermore, a comparative review highlights notable discrepancies between national care standards and WHO recommendations regarding drug selection, dosage, and treatment length for common infections. Together, these data points offer a critical look at the alignment of local prescribing practices with international health guidelines. Fifty “Access” antibiotics are not available in the country, of which 9 are included in the WHO EML/EMLc lists.

The registration of medicines can only be initiated by the manufacturer or its representative, without any incentives provided to assist authorization and future sales of narrow-spectrum antimicrobials, which would be essential to limit selection pressure (from the perspective of the pathogens) and collateral damage to the microbiota of the host (from the patient’s perspective) [27]. Another policy that may contribute to the suboptimal use of “Access” antibiotics is the NSoCs, which do not list them as the first treatment option in many instances and in general recommend for antibiotic prescribing more often than the WHO recommendations do.

A previous study showed that overall antibiotic consumption in Kazakhstan declined over the period 2019–2023, with the proportion of “Access” antibiotics also decreasing from 52.21% in 2019 to 44.33% in 2023 [28]. This observation is consistent with reports from the WHO Regional Office for Europe on the consumption of antimicrobial drugs for the period 2015–2023, which indicate that total antibiotic consumption declined from 17.0 DID in 2015 to 11.6 DID in 2023, while the share of “Access” antibiotics in Kazakhstan remained below the 60% target [29,30]. Two “Watch” antibiotics (azithromycin and ciprofloxacin) were the most commonly consumed antibiotics in the country, and together with ceftriaxone (another “Watch” antibiotic), they accounted for nearly 40% of total consumption [28]. Amoxicillin and its combination with clavulanic acid were the third and fourth most commonly consumed antibiotics [29,30].

The reason for azithromycin being the most consumed antibiotic in Kazakhstan is likely due to the earlier perception of its benefits that arose during the COVID-19 pandemic and still persists to date [28]. The reasons for ciprofloxacin being the second most consumed antibiotic are not entirely clear, but regulatory agencies, including the European Medicines Agency and the U.S. Food and Drug Administration, have issued safety warnings and usage restrictions (i.e., “black box” warnings) for fluoroquinolones due to the risk of serious, potentially disabling adverse effects [31,32]. Of interest is the fact that a study from neighboring China also reported declining trends in “Access” antibiotic consumption, although the core of the most commonly used antibiotics differed from what was observed in this study [33].

The present study is in line with the findings of previous studies, showcasing a declining trend in the overall consumption of “Access” antibiotics from 7.20 DID in 2017 to 5.26 DID in 2023. However, in 2024, this declining trend was reversed, and the consumption of “Access” antibiotics increased to 7.44 DID, exceeding the level observed in 2017. This is consistent with an earlier study on antibiotic consumption in primary care, which confirmed that the overall rate of antibiotic consumption increased in 2024 following a previous decline, including the consumption of “Access” antibiotics [34]. This increase could be driven by several factors, including infectious disease outbreaks, procurement policy changes, or other system-level anomalies. However, analysis of the epidemiological situation does not support the occurrence of infectious disease outbreaks that could explain the observed rise in antibiotic consumption, and no clear changes in procurement policies were identified during this period. Still, media reports suggest increased sales of antibiotics in community pharmacies, which reportedly doubled in 2024 compared to 2023, with most antibiotics being imported [35,36]. One possible explanation for the observed market pattern could be global shortages of certain “Access” antibiotics, particularly amoxicillin and amoxicillin–clavulanic acid, which were reported during the post-COVID period due to increased demand, resurgence of respiratory infections, supply-chain disruptions, and shortages of active pharmaceutical ingredients [37,38].

The Kazakhstan national data on antibiotic consumption should be interpreted with regard to the reported resistance rates. However, there is a paucity of Kazakhstani studies reporting antibiotic susceptibility and resistance patterns, with the majority of studies coming from separate hospital settings. For example, a study by Viderman et al. reported resistance to third-generation cephalosporins and carbapenems as being the most frequent among intensive care unit patients hospitalized in a tertiary-level facility [39]. Another study by Viderman et al. conducted in the same setting reported that resistance to aminoglycosides was the most predominant, followed by resistance to third-generation cephalosporins [40].

Kazakhstan has made substantial efforts to address the problem of emerging AMR by developing antimicrobial resistance and antimicrobial consumption surveillance systems [41], adopting a NAP [42], introducing AMS programs in selected healthcare facilities [43], and restricting over-the-counter sales of antibiotics through the introduction and enforcement of relevant legislation [44]. The current NAP, developed for the period 2023–2027, outlines a set of activities aimed at optimizing the use of antimicrobial agents. One of these activities includes the development of NSoCs for the rational use of antimicrobials in the treatment of common infections, while another envisages the establishment of a national network of healthcare organizations to monitor antimicrobial use and consumption [42]. However, the NAP does not include activities specifically aimed at improving the availability of “Access” antibiotics, nor does it address the need to revise NSoCs to promote broader prescribing of “Access” antibiotics at the primary care level, although such measures could play a critical role in aligning antibiotic use with WHO recommendations [43]. In addition, having a NAP alone, without dedicated funding allocation, may limit the implementation capacity of specific initiatives outlined in the plan [45].

Much remains to be done to improve the national AMS program, beyond refining the NAP. In addition to the limited availability of certain antibiotics, several medicines included in the WHO EML/EMLc lists remain unavailable in the country [46]. There are several possible explanations of the lack of registration of some antibiotics in the country. This is a critical issue as the state cannot independently register essential “Access” antibiotics. Manufacturers may perceive a lack of financial incentives to register some products in the country. Introducing fee waivers or financial incentives for the registration of essential “Access” antibiotics may help offset limited commercial returns [47]. Guaranteed procurement mechanisms may also create predictable demand, making market entry economically viable for manufacturers [48]. Regional pooled procurement across Central Asian countries could increase market size to improve the commercial viability of “Access” antibiotics. Strategic investment in local production of essential antibiotics may provide long-term supply security [49]. Temporary importation mechanisms for non-registered essential antibiotics and the preparation of a legislative framework that would allow for this to work sustainably may also be implemented to mitigate immediate access gaps [50].

Another important policy priority is the reconsideration of NSoCs for common infections, with better alignment to international evidence and a stronger emphasis on prescribing “Access” antibiotics [51]. Further improvements could be achieved by increasing the availability of rapid diagnostic tests (e.g., for group A streptococcal pharyngitis, influenza) for identifying bacterial infections, where administration of antibiotics is indeed warranted [52]. In addition, there is a need to strengthen healthcare providers’ knowledge of evolving AMR, and to promote more rational antibiotic prescribing practices [53].

Digital health technologies (DHTs) have recently emerged as a promising tool to combat AMR and support AMS. DHT may enhance surveillance systems through real-time data collection and rapid identification of resistant strains of public health importance, thereby enabling timely public health responses [54]. DHTs also facilitate access to clinical decision support systems through both mobile applications [55] and software platforms [56], promoting rational antibiotic prescribing by providing rapid access to the best available evidence. Kazakhstan has introduced electronic health record systems and digitalized many healthcare processes; however, the area of AMS remains largely undigitalized, limiting the potential to leverage DHT for optimizing antibiotic use [57].

Despite being the first study analyzing the consumption of “Access” antibiotics based on a large nationwide dataset covering an eight-year period and examining national health policies related to their availability and use, this study has several limitations that need to be acknowledged. First, the consumption data sourced from Vi-ORTIS reflect sales and distribution volumes rather than actual patient-level consumption. Given the possibility of stockpiling in hospital settings, wastage, delayed use, and the possibility for non-human use, the reported figures may not always accurately reflect the amounts of antibiotics that were ultimately prescribed or used by patients, which could have affected the estimates in certain time periods. In alignment with GLASS-AMC, these figures should be regarded as estimates of antibiotic availability for human use and, in this sense, may approximate antibiotic consumption; however, they do not fully substitute for detailed data on prescribing, dispensing, or administration. Second, the data available for analysis did not contain patient-level information; incorporating such data would allow for a better understanding of which patient groups are more or less likely to receive “Access” antibiotics and would provide deeper insight into prescribing practices. Third, the data lack disaggregation by province, making it difficult to identify regions with disproportionately low consumption of “Access” antibiotics. Also, the study assumes that all sold or distributed antibiotics were consumed by humans, which may not necessarily be the case. Finally, some relevant policy documents within the scope of this study may not have been identified or analyzed. Nevertheless, the study highlights critical policy gaps affecting the availability and use of “Access” antibiotics in a middle-income country, providing a foundation for strengthening AMS policies.

## 4. Materials and Methods

### 4.1. Study Hypothesis

This study is based on the assumption that there is a strong link between health policies and antibiotic use in Kazakhstan, which is supported by several observations. First, the country’s healthcare system is hierarchically structured and centrally regulated, with the MoH serving as the primary decision-making body, while regional health authorities have limited autonomy in matters related to pharmaceutical regulation, procurement, and the development of clinical standards of care [58]. Second, the marketing and sale of pharmaceuticals that have not undergone state registration in Kazakhstan, or whose registration validity has expired, are prohibited and strictly regulated by the government [59]. Third, the NSoCs are widely used throughout the country as standardized instruments for clinical decision-making and are integrated into the broader framework governing the provision and reimbursement of healthcare services within the state-funded healthcare system [57]. Taken together, these observations suggest that national regulatory and clinical policies may substantially influence antibiotic availability and patterns of antibiotic consumption across the country.

### 4.2. Study Design

This study employed a retrospective, observational design combining both quantitative and qualitative methodologies. The quantitative component consisted of a pharmacoepidemiological analysis of a large dataset of pharmaceuticals classified under the J01 ATC group (antibacterials for systemic use), obtained from Vi-ORTIS, a national pharmaceutical market research company. The qualitative component involved a desk review of legislative acts regulating the availability and registration of pharmaceuticals in Kazakhstan, as well as an analysis of national standards of care in comparison with the WHO EML/EMLc lists [21] and the AWaRe Antibiotic Book [26]. The following subsections provide a more detailed description of the study methodology.

### 4.3. Quantitative Study

Vi-ORTIS is the largest pharmaceutical market research company based in Kazakhstan, collecting data on all pharmaceuticals sold or distributed in the country across both ambulatory and hospital healthcare sectors. Data collection in the ambulatory sector is conducted using the “PharmCenter” software developed by Vi-ORTIS, which is provided free of charge to community pharmacies [60]. In the hospital sector, Vi-ORTIS obtains data from SK-Pharmacia, the sole distributor of pharmaceuticals to hospital facilities in Kazakhstan [61].

Vi-ORTIS data are frequently used in pharmacoepidemiological research in the Kazakhstani context [28,34,62]. Access to the database is provided on a subscription basis. For this study, data on all pharmaceuticals belonging to the J01 group were obtained for the period from 1 January 2017 to 31 December 2024 at the ATC level 5 (substance level).

To convert the data on J01 pharmaceuticals sold or distributed in Kazakhstan, the methodology outlined in the Global Antimicrobial Resistance and Use Surveillance System (GLASS-AMC) was strictly followed [63]. Data on active substances, trade names, dosage forms, package sizes, routes of administration, level of care (primary vs. hospital), and the number of packages sold or distributed, extracted from the Vi-ORTIS portal, were entered into the GLASS-AMC template. This enabled the calculation of defined daily doses per 1000 inhabitants per day (DIDs) for each ATC level 5 substance [64]. The GLASS-AMC template includes built-in data checking and validation functions, which were used to ensure the accuracy of the DID calculations.

The GLASS-AMC template generates output in the form of DIDs for each ATC level 5 substance, stratified by primary and hospital care settings on an annual basis [65]. In the following step, all ATC level 5 substances were grouped into four categories (Access, Watch, Reserve, and Not classified) according to the WHO AWaRe classification [8]. Subsequent analyses primarily focused on antibiotics belonging to the “Access” group.

### 4.4. Qualitative Study

The qualitative component of the study was conducted using content analysis of key provisions outlined in legislative acts retrieved from the Adilet database (a centralized repository of all legislative acts adopted in the country that is maintained by the Ministry of Justice) [66], as well as from the database of national standards of care maintained by the National Research Center for Healthcare Development (NRCHD) named after S. Kairbekova [67]. Searches in both databases were performed in Kazakh and Russian since health policy documents in Kazakhstan may be published in either language. All searches were performed independently by two reviewers (L.M. and N.A.), and any disagreements regarding the relevance of policy documents for inclusion in the study were resolved through consultation with a third reviewer (Y.S.). Overall, 21 NSoCs were identified and their full texts were reviewed and analyzed. Of these, 10 documents were duplicates published in both Kazakh and Russian languages, while one document represented an outdated version of the NSoC on acute sinusitis for which a newer version was available. After excluding duplicates and the outdated document, a total of 10 NSoCs were included in the final analysis.

For searches in the Adilet database conducted in Russian, the following queries were used: (i) “лекарственный фoрмуляр” (drug formulary), (ii) “регистрация” (registration), (iii) “ре-регистрация” (re-registration), (iv) “лекарственные средства” (pharmaceuticals), and (v) “медикаменты” (medicines). For searches in Kazakh, the following keywords were used: (i) “дәрілік фoрмуляр” (drug formulary), (ii) “тіркеу” (registration), (iii) “қайта тіркеу” (re-registration), (iv) “дәрілер” (pharmaceuticals), and (v) “дәрі-дәрмектер” (medicines). As a first step, the titles of all identified legislative acts were screened, and relevant documents were recorded in an MS Excel (Microsoft Corp. Redmond, WA, USA) spreadsheet. Subsequently, the full texts of the selected legislative acts were reviewed and analyzed using predefined analytical categories. Information extracted from the documents included provisions related to medicine registration, re-registration, and inclusion in the national EML, referred to in Kazakhstan as the “drug formulary.” The information obtained from the documents was then organized into tables.

For the analysis of key provisions within the national standards of care governing antibiotic prescribing for common infections in primary care, the WHO AWaRe Antibiotic Book was used as a reference. First, a list of common infections was compiled based on this source [26]. Next, the NSoC database available on the NRCHD website was reviewed [67], and for each infection listed in the WHO AWaRe Antibiotic Book, the corresponding national standard of care was identified. Predefined analytical categories were used to extract information related to antibiotic prescribing, including infection type, recommended antibiotics, indications for antibiotic use, dosage, and duration of therapy. The extracted information was organized into comparative tables, while the AWaRe classification group for each antibiotic was retrieved from the latest version of the classification [68] and added to the tables.

### 4.5. Definitions Used

In this study, healthcare sectors refer to different healthcare settings in which antibiotics were distributed and consumed. Specifically, the community sector refers to outpatient (ambulatory and retail pharmacy) settings, while the hospital sector refers to inpatient healthcare facilities. The total sector represents the combined consumption across both settings.

The term “unavailable” refers to antibiotics that are not accessible in the national context due to regulatory or market-related constraints. More specifically, an antibiotic was considered unavailable if it was not registered in the country, not included in the national formulary or not present on the pharmaceutical market during the study period.

### 4.6. Data Analysis

The DID for each ATC5 code within the “Access” group of antibiotics was calculated for each year of the study period (2017–2024) using the GLASS-AMC template. These calculations were performed across the entire healthcare sector (total consumption), as well as separately for the community and hospital sectors. Subsequently, the Statistical Package for the Social Sciences (SPSS version 24.0; IBM Corp., Endicott, NY, USA), was used to evaluate trends in ATC5-level consumption by calculating the APC with 95% CI. The APC with 95% CI was also calculated for the total consumption of all “Access” antibiotics, as well as stratified by community and hospital sectors. Furthermore, the MK test, a non-parametric statistical method was used to detect monotonic trends (consistent upward or downward movement) in antibiotic consumption data. Statistical significance was defined as a *p*-value < 0.05.

### 4.7. Reporting Guidelines

This manuscript adheres to the STROBE (Strengthening the Reporting of Observational Studies in Epidemiology) guidelines for observational studies [69], to ensure methodological rigor, transparency, and reproducibility; the STROBE checklist is provided in Supplementary Material S2.

## 5. Conclusions

In Kazakhstan, “Access” antibiotics constitute less than 60% of consumption recommended by the WHO, and their share declined during the study period, reaching 37.86% in 2024. This study helps to better understand potential policy-related barriers that may influence the availability and use of “Access” antibiotics. A significant proportion of antibiotics listed in the WHO EML remain unavailable in Kazakhstan due to national pharmaceutical registration policies. Reliance on manufacturer-initiated drug registration may contribute to delays or limitations in the inclusion of certain essential antibiotics in the national market. The analysis of national standards of care suggests a tendency to include recommendations for “Watch” antibiotics in some clinical scenarios, which may contribute to their use in routine practice.

A coordinated policy response is needed to strengthen AMS. The absence of “Access” antibiotics creates a structural driver of inappropriate prescribing, effectively undermining AMS efforts and increasing reliance on “Watch” antibiotics. “Access” antibiotics must be considered as a public good, and the results from this analysis suggest that market alone cannot ensure supply: essential, low-cost antibiotics are undersupplied due to limited profitability, necessitating policy intervention, including revising the national frameworks for drug registration and re-registration to improve the availability of “Access” antibiotics, aligning national standards of care with WHO recommendations, and promoting more appropriate prescribing practices.

## Figures and Tables

**Table 1:** WHO AWaRe “Access” antibiotics listed in the EML and/or EMLc that are not available in Kazakhstan.

Substance	ATC5* Code	Pharmacological Group
Dicloxacillin	J01CF01	Beta-lactamase resistant penicillins
Cloxacillin	J01CF02	
Meticillin	J01CF03	
Oxacillin	J01CF04	
Flucloxacillin	J01CF05	
Nafcillin	J01CF06	
Phenoxymethylpenicillin	J01CE02	Beta-lactamase sensitive penicillins
Procaine-benzylpenicillin	J01CE09	
Trimethoprim	J01EA01	Trimethoprim and derivatives

ATC5*—Anatomical Therapeutic Chemical classification, level 5.

**Table 2:** The provisions of the Ministry of Health Order regulating the registration of new drugs.

Provisions	Description
Applicant	Manufacturer or manufacturer’s representative
Registration fee	11 monthly calculation indicators * for primary registration and 5 for re-registration
List of documents submitted by an applicant	(1) An application in the form of an electronic document certified by the applicant’s digital signature; (2) An electronic copy of proof of payment of the registration fee; (3) An electronic copy of the conclusion on the safety, quality, and efficacy of the drug issued by a state expert organization.

* As of 2026, one monthly calculation indicator is equivalent to 4325 national currency units, or approximately 8.65 US dollars.

**Table 3:** Antibiotics recommended by national standards of care for patients with common infections at the primary care level, compared with the recommendations of the WHO AWaRe Antibiotic Book.

Disease	Antibiotic Recommended by the National Standards of Care (AWaRe * Group)	Antibiotic Recommended by the WHO Antibiotic Book (AWaRe Group)
Acute sinusitis	Ampicillin (“Access”)	Amoxicillin (“Access”)
	Amoxicillin (“Access”)	Amoxicillin + clavulanate (“Access”)
	Amoxicillin + clavulanate (“Access”)	
	Azithromycin (“Watch”)	
Community-acquired pneumonia	Amoxicillin (“Access”)	Amoxicillin (“Access”)
	Amoxicillin + clavulanate (“Access”)	Amoxicillin + clavulanate (“Access”)
	Azithromycin (“Watch”)	Phenoxymethylpenicillin (“Access”)
		Doxycycline (“Access”)
		Cefotaxime (“Watch”)
		Ceftriaxone (“Watch”)
		Clarithromycin (“Watch”)
Chronic bronchitis/COPD **	Amoxicillin + clavulanate (“Access”)	Amoxicillin (“Access”)
	Cefuroxime (“Watch”)	Cefalexin (“Access”)
	Cefixime (“Watch”)	Doxycycline (“Access”)
	Azythromycin (“Watch”)	Amoxicillin + clavulanate (“Access”)
	Ceftriaxone (“Watch”)	
Otitis media	Ampicillin (“Access”)	Amoxicillin (“Access”)
	Amoxicillin + clavulanate (“Access”)	Amoxicillin + clavulanate (“Access”)
	Azithromycin (“Watch”)	
Pharyngitis	Antibiotics are not indicated	Amoxicillin (“Access”)
		Phenoxymethylpenicillin (“Access”)
		Cefalexin (“Access”)
		Clarithromycin (“Watch”)
Ulcerative gingivitis	Tinidazole (“Access”)	Amoxicillin (“Access”)
		Phenoxymethylpenicillin (“Access”)
Periodontitis	Doxycycline (“Access”)	Amoxicillin (“Access”)
	Tinidazole (“Access”)	Phenoxymethylpenicillin (“Access”)
Lower urinary tract infections: cystitis and urethritis	Levofloxacin (“Watch”)	Amoxicillin + clavulanate (“Access”)
	Ciprofloxacin (“Watch”)	Nitrofurantoin (“Access”)
	Amoxicillin + clavulanate (“Access”)	Trimethoprim (“Access”)
	Cefixime (“Watch”)	Sulfamethoxazole + trimethoprim (“Access”)
	Fosfomycin (“Watch”)	
	Furazidin (“Access”)	
	Nitrofurantoin (“Access”)	
	Ofloxacin (“Watch”)	
Impetigo	Amoxicillin (“Access”)	Amoxicillin + clavulanate (“Access”)
	Cefazolin (“Watch”)	Cloxacillin (“Access”)
	Ceftriaxone (“Watch”)	Cefalexin (“Access”)
	Erythromycin (“Watch”)	
	Azithromycin (“Watch”)	
	Clarithromycin (“Watch”)	
	Doxycycline (“Access”)	
	Ciprofloxacin (“Watch”)	
	Levofloxacin (“Watch”)	
	Ofloxacin (“Watch”)	
	Gentamicin (“Access”)	
Skin abscess, boil and carbuncle	Amoxicillin + clavulanate (“Access”)	Amoxicillin + clavulanate (“Access”)
	Cefalexin (“Access”)	Cloxacillin (“Access”)
	Cefuroxime (“Watch”)	Cefalexin (“Access”)
	Erythromycin (“Watch”)	
	Oxacillin (“Access”)	
	Cefazolin (“Watch”)	
	Ceftriaxone (“Watch”)	

* AWaRe—Access, Watch, Reserve; ** COPD—chronic obstructive pulmonary disease; Shaded areas indicate antibiotics that are recommended by the national standards of care, but are not recommended by the WHO AWaRe Antibiotic Book.

**Table 4:** Antibiotics recommended by the WHO AWaRe Antibiotic Book for the treatment of patients with common infections at the primary care level that are not available in Kazakhstan.

Antibiotic Recommended by the WHO Antibiotic Book That Is Not Available in Kazakhstan	AWaRe * Group
Phenoxymethylpenicillin	“Access”
Trimethoprim	
Cloxacillin	

* AWaRe—Access, Watch, Reserve.

## Data Availability

The data (Data S1) presented in this study are provided as Supplementary Materials.

## Supplementary Materials

The following supporting information can be downloaded at https://www.mdpi.com/article/10.3390/antibiotics15060585/s1, Table S1: Total consumption of the WHO AWaRe Access group antibiotics; Table S2: Consumption of the WHO AWaRe Access group antibiotics in the community sector; Table S3: Consumption of the WHO AWaRe Access group antibiotics in the hospital sector; Table S4: WHO AWaRe Access group antibiotics that are not listed in the essential medicines list and/or essential medicines list for children and are not available in Kazakhstan; Table S5: Comparison of national standards of care for common infections at the primary care level with WHO AWaRe Antibiotic Book recommendations; Table S6: STROBE checklist; Data S1: Minimal Dataset.

## Abbreviations

The following abbreviations are used in this manuscript:

APS	Annual Percent Change
AMR	Antimicrobial Resistance
AMS	Antimicrobial Stewardship
ATC	Anatomical Therapeutic Chemical Classification
AWaRe	Access, Watch, Reserve
COPD	Chronic obstructive pulmonary disease
DDD	Defined Daily Doses
DHT	Digital Health Technologies
DID	Defined Daily Doses per 1000 Inhabitants per Day
EML	Essential Medicines List
GLASS-AMC	Global Antimicrobial Resistance and Use Surveillance System
LMICs	Low- and Middle-Income Countries
M-K	Mann–Kendall
MoH	Ministry of Health
NAP	National Action Plan
NSoC	National standards of care
NRCHD	National Research Center for Healthcare Development
R&D	Research and development
SPSS	Statistical Package for the Social Sciences
WHO	World Health Organization
UNGA	United Nations General Assembly
95% CI	95% Confidence Interval

## References

[1] Salam M.A., Al-Amin M.Y., Salam M.T., Pawar J.S., Akhter N., Rabaan A.A., Alqumber M.A.A. (2023). Antimicrobial Resistance: A Growing Serious Threat for Global Public Health. Healthcare.

[2] GBD 2021 Antimicrobial Resistance Collaborators (2024). Global burden of bacterial antimicrobial resistance 1990–2021: A systematic analysis with forecasts to 2050. Lancet.

[3] Uddin T.M., Chakraborty A.J., Khusro A., Zidan B.M.R.M., Mitra S., Emran T.B., Dhama K., Ripon M.K.H., Gajdács M., Sahibzada M.U.K. (2021). Antibiotic resistance in microbes: History, mechanisms, therapeutic strategies and future prospects. J. Infect. Public Health.

[4] World Health Organization Global Antibiotic Resistance Surveillance Report 2025. https://www.who.int/publications/i/item/9789240116337.

[5] Muteeb G., Rehman M.T., Shahwan M., Aatif M. (2023). Origin of Antibiotics and Antibiotic Resistance, and Their Impacts on Drug Development: A Narrative Review. Pharmaceuticals.

[6] Khadse S.N., Ugemuge S., Singh C. (2023). Impact of antimicrobial stewardship on reducing antimicrobial resistance. Cureus.

[7] Figueras A. (2024). A Global Call to Action on Antimicrobial Resistance at the UN General Assembly. Antibiotics.

[8] Zanichelli V., Sharland M., Cappello B., Moja L., Getahun H., Pessoa-Silva C., Sati H., van Weezenbeek C., Balkhy H., Simão M. (2023). The WHO AWaRe (Access, Watch, Reserve) antibiotic book and prevention of antimicrobial resistance. Bull. World Health Organ..

[9] Gandra S., Kotwani A. (2019). Need to improve availability of “access” group antibiotics and reduce the use of “watch” group antibiotics in India for optimum use of antibiotics to contain antimicrobial resistance. J. Pharm. Policy Pract..

[10] World Health Organization Antibiotic Use: Target ≥70% of Total Antibiotic Use Being Access Group Antibiotics (70% Access Target). https://www.who.int/data/gho/indicator-metadata-registry/imr-details/5767.

[11] Mulchandani R., Tiseo K., Nandi A., Klein E., Gandra S., Laxminarayan R., Van Boeckel T. (2025). Global trends in inappropriate use of antibiotics, 2000–2021: Scoping review and prevalence estimates. BMJ Public Health.

[12] Zanichelli V., Monnier A.A., Gyssens I.C., Adriaenssens N., Versporten A., Pulcini C., Le Maréchal M., Tebano G., Vlahovic-Palcevski V., Stanic Benic M. (2018). Variation in antibiotic use among and within different settings: A systematic review. J. Antimicrob. Chemother..

[13] Lim J.M., Singh S.R., Duong M.C., Legido-Quigley H., Hsu L.Y., Tam C.C. (2020). Impact of national interventions to promote responsible antibiotic use: A systematic review. J. Antimicrob. Chemother..

[14] Kizito M., Lalitha R., Kajumbula H., Muhumuza R., Kintu M.G., Muyanja D., Byakika-Kibwika P. (2024). ‘Some patients demand for a prescription of an antibiotic’: An assessment of barriers and facilitators to rational antimicrobial use in a private health facility in Uganda. JAC Antimicrob. Resist..

[15] Semenova Y., Akhmetova K., Semenov D., Makalkina L., Surov V., Pivina L., Turgambayeva A., Belikhina T., Maukayeva S., Goremykina M. (2025). Social Determinants of Health and Antibiotic Consumption. Antibiotics.

[16] Sartelli M., Hardcastle T.C., Catena F., Chichom-Mefire A., Coccolini F., Dhingra S., Haque M., Hodonou A., Iskandar K., Labricciosa F.M. (2020). Antibiotic Use in Low and Middle-Income Countries and the Challenges of Antimicrobial Resistance in Surgery. Antibiotics.

[17] Bebell L.M., Muiru A.N. (2014). Antibiotic use and emerging resistance: How can resource-limited countries turn the tide?. Glob. Heart.

[18] Cabral C., Zhang T., Oliver I., Little P., Yardley L., Lambert H. (2024). Influences on use of antibiotics without prescription by the public in low- and middle-income countries: A systematic review and synthesis of qualitative evidence. JAC Antimicrob. Resist..

[19] Glushkova N., Semenova Y., Sarria-Santamera A. (2023). Editorial: Public health challenges in post-Soviet countries during and beyond COVID-19. Front. Public Health.

[20] Adilet Order of the Ministry of Health of the Republic of Kazakhstan Approving the National Drug Formulary of the Republic of Kazakhstan (Order No. ҚР ДСМ–41). https://adilet.zan.kz/rus/docs/V2100022782/history.

[21] World Health Organization Model Lists of Essential Medicines. https://www.who.int/groups/expert-committee-on-selection-and-use-of-essential-medicines/essential-medicines-lists.

[22] Adilet Order of the Ministry of Health of the Republic of Kazakhstan Approving the Rules for Registration and Re-Registration of Drugs or Medical Devices. https://adilet.zan.kz/rus/docs/V2100022175.

[23] National Bank of the Republic of Kazakhstan Official Foreign Exchange Rates on Average for the Period. https://nationalbank.kz/en/news/oficialnye-kursy.

[24] Katsaga A., Kulzhanov M., Karanikolos M., Rechel B. (2012). Kazakhstan: Health system review. Health Syst. Transit..

[25] MedElement National Standards of Care. https://diseases.medelement.com/?searched_data=diseases&q=&mq=&tq=&diseases_filter_type=list&diseases_content_type=4&section_medicine=0&category_mkb=0&parent_category_mkb=0.

[26] World Health Organization The WHO AWaRe (Access, Watch, Reserve) Antibiotic Book. https://www.who.int/publications/i/item/9789240062382.

[27] Elbehiry A., Marzouk E., Abalkhail A. (2025). Antimicrobial resistance at a turning point: Microbial drivers, One Health, and global futures. Front. Microbiol..

[28] Semenova Y., Yergaliyeva A., Aimurziyeva A., Manatova A., Kuntuganova A., Makalkina L., Aldiyarova N., Semenov D., Lim L. (2024). A Nationwide Evaluation of Antibiotic Consumption in Kazakhstan from 2019 to 2023. Antibiotics.

[29] World Health Organization Regional Office for Europe (2024). Antimicrobial Medicines Consumption (AMC) Network: AMC Data 2022.

[30] World Health Organization Regional Office for Europe Antimicrobial Medicines Consumption (AMC) Network: AMC Data 2023. https://www.who.int/europe/publications/i/item/9789289061872.

[31] European Medicines Agency Fluoroquinolone Antibiotics: Reminder of Measures to Reduce the Risk of Long-Lasting, Disabling and Potentially Irreversible Side Effects. https://www.ema.europa.eu/en/news/fluoroquinolone-antibiotics-reminder-measures-reduce-risk-long-lasting-disabling-potentially-irreversible-side-effects.

[32] McCarthy M. (2016). US warns against use of fluoroquinolones for uncomplicated infections. BMJ.

[33] Li W., Du F., Xiao R., Ko W., Chen S., Zhang J., Wu S., Zheng B., Shi L., Guan X. (2026). Present trajectories to forecasted future: A longitudinal analysis of hospital antibiotic use in China from 2013 to 2024 and projections to 2030. Int. J. Antimicrob. Agents.

[34] Akhmetova K., Makalkina L., Pivina L., Lim L., Aukenov N., Boranbayev K., Stukas R., Belikhina T., Aldiyarova N., Turgambayeva A. (2025). Trends in ‘Watch’ and ‘Reserve’ Antibiotic Use in Primary Care in Kazakhstan: The Imperative for Enhancing Stewardship Strategies. Antibiotics.

[35] 24KZ Kazakhstanies Started Purchasing Antibiotics More Often. https://24.kz/ru/news/social/672406-kazakhstantsy-stali-chashche-pokupat-antibiotiki.

[36] Ranking.KZ The Consumption of Antibiotics Has Increased in Kazakhstan. https://ranking.kz/digest/industries-digest/v-kazahstane-vyroslo-potreblenie-antibiotikov.html.

[37] Otsubo Y., Matsunaga N., Tsukada A., Kaneko T., Isobe Y., Horikoshi Y. (2025). Chronic amoxicillin shortage led to alternative broad-spectrum antimicrobial use in pediatric clinics. J. Infect. Chemother..

[38] Pagano F., De Marco G., Trojano B., Amato C., Micillo M., Cecere G., Guarino A., Lo Vecchio A. (2025). Impact of Amoxicillin Shortage on Pediatric Antibiotic Prescriptions in Primary Care. Antibiotics.

[39] Viderman D., Khamzina Y., Kaligozhin Z., Khudaibergenova M., Zhumadilov A., Crape B., Azizan A. (2018). An observational case study of hospital-associated infections in a critical care unit in Astana, Kazakhstan. Antimicrob. Resist. Infect. Control.

[40] Viderman D., Brotfain E., Khamzina Y., Kapanova G., Zhumadilov A., Poddighe D. (2019). Bacterial resistance in the intensive care unit of developing countries: Report from a tertiary hospital in Kazakhstan. J. Glob. Antimicrob. Resist..

[41] National Center for Public Health of the Ministry of Health of the Kazakhstan Republic Tracking AMR Country Self-Assessment Survey (TrACSS). https://hls.kz/wp-content/uploads/2025/09/Tracking-AMR-Country-Self-Assessment-Survey-TrACSS-1.pdf.

[42] National Center for Public Health of the Ministry of Health of the Kazakhstan Republic Roadmap: On Measures to Contain Antimicrobial Resistance in the Kazakhstan Republic for 2023–2027. https://hls.kz/ru/piik-ru.

[43] World Health Organization Mainstreaming Antimicrobial Resistance Interventions into Primary Health Care in Kazakhstan: Mission Report. https://www.who.int/europe/publications/i/item/WHO-EURO-2026-12609-52383-80741.

[44] Zarpullayev N.Y., Yurkovskaya O.A., Mukanova D.A., Kozhakhmetova D.K., Botabayeva A.S., Smail Y.M., Shamshudinov T.M., Kussainova A.A., Derbissalina G.A., Urazalina N.M. (2025). Analysis of antibiotic prescribing practices in primary health care settings. Part I. Nauka I Zdr..

[45] Chua A.Q., Verma M., Hsu L.Y., Legido-Quigley H. (2021). An analysis of national action plans on antimicrobial resistance in Southeast Asia using a governance framework approach. Lancet Reg. Health West. Pac..

[46] Shalabayeva D., Orazova G., Zhussupova G. (2022). Comparative analysis of 21 lists of essential medicines of the World Health Organization with the Kazakhstan National Medicinal Formulary. J. Health Dev..

[47] Otaigbe I.I. (2025). Mitigating inequitable access to appropriate antibiotics in low- and middle-income countries. JAC Antimicrob. Resist..

[48] Leão R., Ijatuyi E.J., Goulao L.F. (2023). How Public Procurement Mechanisms Can Be Used as a Tool for Developing Pro-Poor Food Value Chains: From Entry Points to Interventions. Sustainability.

[49] Husain L., Hu Y., Huang Y. (2024). Towards mission-driven investment in new antimicrobials? What role for Chinese strategic industrial financing vehicles in responding to the challenge of antimicrobial resistance?. Glob. Health.

[50] Lőrinczy L., Turbucz B., Hankó B., Zelkó R. (2023). Managing Antibiotic Shortages in Inpatient Care—A Review of Recent Years in Comparison with the Hungarian Status. Antibiotics.

[51] Knowles R., Chandler C., O’Neill S., Sharland M., Mays N. (2024). A systematic review of national interventions and policies to optimize antibiotic use in healthcare settings in England. J. Antimicrob. Chemother..

[52] Alatawi A.D., Hetta H.F., Ali M.A.S., Ramadan Y.N., Alaqyli A.B., Alansari W.K., Aldhaheri N.H., Bin Selim T.A., Merdad S.A., Alharbi M.O. (2025). Diagnostic Innovations to Combat Antibiotic Resistance in Critical Care: Tools for Targeted Therapy and Stewardship. Diagnostics.

[53] Semenova Y., Akhmetova K., Rakhmatullaeva S., Muminova M., Fakhriddinova D., Dzhusupov K., Kanymetova A., Ashyralieva D., Saidova M., Yakubova S. (2026). Antibiotic Resistance Awareness and Prescribing Behavior Among General Practitioners in Kazakhstan, Kyrgyzstan, Uzbekistan, and Tajikistan. Antibiotics.

[54] Donelle L., Comer L., Hiebert B., Hall J., Shelley J.J., Smith M.J., Kothari A., Burkell J., Stranges S., Cooke T. (2023). Use of digital technologies for public health surveillance during the COVID-19 pandemic: A scoping review. Digit. Health.

[55] Roganović J., Djordjević S., Barać M., Crnjanski J., Milanović I., Ilić J. (2024). Dental Antimicrobial Stewardship: Developing a Mobile Application for Rational Antibiotic Prescribing to Tackle Misdiagnosis. Antibiotics.

[56] Xu R., Wu L., Wu L., Xu C., Mu T. (2023). Effectiveness of decision support tools on reducing antibiotic use for respiratory tract infections: A systematic review and meta-analysis. Front. Pharmacol..

[57] Ministry of Health Digital Journey: Kazakhstan’s Healthcare. https://www.gov.kz/memleket/entities/dsm/press/article/details/4848.

[58] Semenova Y., Lim L., Salpynov Z., Gaipov A., Jakovljevic M. (2024). Historical evolution of healthcare systems of post-soviet Russia, Belarus, Kazakhstan, Kyrgyzstan, Tajikistan, Turkmenistan, Uzbekistan, Armenia, and Azerbaijan: A scoping review. Heliyon.

[59] Adilet Code of the Republic of Kazakhstan “On Public Health and the Healthcare System”. https://adilet.zan.kz/rus/docs/K2000000360.

[60] Vi-ORTIS. Market Research Company. https://base.viortis.kz/Account/LogOn?ReturnUrl=%2f.

[61] (2024). SK-Pharmacia. https://sk-pharmacy.kz/rus/.

[62] Semenova Y., Kussainova A., Kassym L., Aimurziyeva A., Semenov D., Makalkina L., Aldiyarova N., Avdeyev A., Lim L. (2025). Systemic antiviral consumption in Kazakhstan. Sci. Rep..

[63] World Health Organization (2020). GLASS Manual on the Management of Antimicrobial Consumption Data.

[64] Norwegian Institute of Public Health ATC/DDD Index. https://atcddd.fhi.no/atc_ddd_index/?code=J&showdescription=yes.

[65] World Health Organization The ATC/DDD Methodology. https://www.who.int/tools/atc-ddd-toolkit/methodology.

[66] Adilet. https://adilet.zan.kz/kaz/.

[67] Website of National Research Center of Healthcare Development Named After S. Kairbekova, Ministry of Healthcare. https://nrchd.kz/.

[68] World Health Organization AWaRe Classification of Antibiotics for Evaluation and Monitoring of Use, 2023. https://www.who.int/publications/i/item/WHO-MHP-HPS-EML-2023.04.

[69] von Elm E., Altman D.G., Egger M., Pocock S.J., Gøtzsche P.C., Vandenbroucke J.P. (2007). Strengthening the Reporting of Observational Studies in Epidemiology (STROBE) statement: Guidelines for reporting observational studies. BMJ.

