# Supplementary material for: How Health Policies Shape Antibiotic Use: A Nationwide Study of “Access”-Group Antibiotics Availability and Consumption in a Middle-Income Country: The Case of Kazakhstan
Source: Antibiotics (Basel). 2026 Jun 8;15(6):585. doi: 10.3390/antibiotics15060585 (PMC13296338; doi:10.3390/antibiotics15060585)
Supplement: Supplementary file 1 [file antibiotics-15-00585-s001.zip › supplementary tables.pdf]

Table S1. Total consumption of the WHO AWaRe Access group antibiotics.

Substance ([Ω]ATC5 code)	Pharmacological subgroup (ATC3 code)	DID[α]								APC[0]	Trend analysis [a]
		2017	2018	2019	2020	2021	2022	2023	2024	(95% CI*)	
Doxycycline (J01AA02)	Tetracyclines (J01A)	0.555	0.458	0.490	0.512	0.457	0.417	0.501	0.749	2.34% (-4.43; 9.60)	n.s.** (p=0.548)
Tetracycline (J01AA07)		0.185	0.185	0.076	0.081	0.113	0.087	0.144	0.068	-8.54% (-20.29; 4.94)	n.s. (p=0.138)
Chloramphenicol (J01BA01)	Amphenicols (J01B)	0.338	0.331	0.322	0.287	0.285	0.249	0.268	0.416	-0.44% (-6.75; 6.31)	n.s. (p=0.089)
Thiamphenicol (J01BA02)		0.000	0.000	0.000	0.000	0.000	0.000	0.000	0.006	-	-
Ampicillin (J01CA01)	Beta-lactam antibacterials, penicillins (J01C)	0.632	0.522	0.446	0.233	0.343	0.248	0.277	0.287	-11.29% (-18.96; -2.89)	Significant decrease (p=0.044)
Amoxicillin (J01CA04)		1.146	1.064	1.270	1.154	0.836	0.700	1.053	1.488	0.40% (-9.53; 9.66)	n.s. (p=0.452)
Benzylpenicillin (J01CE01)		0.092	0.074	0.031	0.024	0.022	0.046	0.027	0.029	-13.20% (-26.46; 2.46)	n.s. (p=0.086)
Benzathine Benzylpenicillin (J01CE08)		0.000	0.015	0.000	0.000	0.000	0.000	0.000	0.000	-	-
Ampicillin and beta-lactamase inhibitor (J01CR01)		0.000	0.000	0.000	0.000	0.000	0.000	0.003	0.000	-	-
Amoxicillin and beta-lactamase inhibitor (J01CR02)		0.688	0.547	0.408	0.424	0.621	0.736	0.816	1.398	11.46% (-1.03; 25.52)	Significant increase (p=0.033)
Sultamicillin (J01CR04)		0.000	0.000	0.000	0.000	0.000	0.000	0.000	0.000	-	-
Cefalexin (J01DB01)	Other beta-lactam antibacterials (J01D)	0.004	0.002	0.002	0.001	0.000	0.000	0.000	0.000	-	-
Cefazolin (J01DB04)		1.067	1.071	0.889	0.965	0.707	0.676	0.651	0.893	-5.63% (-10.96; 0.02)	Significant decrease (p=0.031)
Sulfaisodimidine (J01EB01)	Sulfonamides and trimethoprim (J01E)	0.066	0.175	0.181	0.200	0.201	0.120	0.000	0.000	-	-
Sulfamethoxazole and trimethoprim (J01EE01)		0.426	0.411	0.305	0.328	0.320	0.288	0.291	0.428	-2.21% (-8.51; 4.52)	n.s. (p=0.199)

Clindamycin (J01FF01)		0.003	0.003	0.002	0.003	0.005	0.005	0.005	0.005	9.73% (1.24; 18.94)	n.s. (p=0.076)
Gentamicin (J01GB03)		0.922	0.861	0.699	0.742	0.365	0.319	0.367	0.135	-21.90% (-29.53; -13.44)	Significant decrease (p=0.002)
Amikacin (J01GB06)		0.107	0.071	0.076	0.081	0.092	0.027	0.040	0.105	-6.96% (-22.82; 12.15)	n.s. (p=0.452)
Metronidazole (J01XD01)		0.336	0.384	0.360	0.405	0.295	0.299	0.343	0.625	3.54% (-5.44; 13.36)	n.s. (p=0.361)
Tinidazole (J01XD02)		0.000	0.000	0.000	0.000	0.000	0.000	0.000	0.000	-	-
Ornidazole (J01XD03)		0.002	0.002	0.001	0.000	0.001	0.001	0.001	0.001	-11.16% (-26.55; 7.46)	n.s. (p=0.238)
Nitrofurantoin (J01XE01)		0.333	0.307	0.277	0.208	0.293	0.291	0.271	0.477	2.88% (-6.00; 12.60)	n.s. (p=0.360)
Furazidin (J01XE03)		0.285	0.274	0.239	0.232	0.167	0.183	0.202	0.332	-1.87% (-10.64; 7.77)	n.s. (p=0.199)
Spectinomycin (J01XX04)		0.008	0.006	0.006	0.001	0.000	0.000	0.000	0.000	-	-

[Ω]ATC5 - Anatomical Therapeutic Chemical classification. [α]DID – Defined Daily Doses per 1000 inhabitants per day. [0]APC – Average Annual Percent Change. *CI – Confidence Interval. [a]Mann-Kendall-test. **n.s. – not significant

Table S2. Consumption of the WHO AWaRe Access group antibiotics in the community sector.

Substance ([Ω]ATC5 code)	Pharmacological subgroup (ATC3 code)	DID[α]								APC[0] (95% CI*)	Trend analysis [a]
		2017	2018	2019	2020	2021	2022	2023	2024		
Doxycycline (J01AA02)	Tetracyclines (J01A)	0.484	0.394	0.470	0.424	0.399	0.382	0.466	0.659	2.79% (-3.79; 9.81)	n.s.** (p=0.548)
Tetracycline (J01AA07)		0.184	0.182	0.075	0.081	0.113	0.087	0.143	0.068	-8.39% (-20.17; 5.13)	n.s. (p=0.138)
Chloramphenicol (J01BA01)	Amphenicols (J01B)	0.334	0.326	0.319	0.283	0.283	0.248	0.267	0.415	-0.27% (-6.60; 6.48)	n.s. (p=0.098)
Thiamphenicol (J01BA02)		0.000	0.000	0.000	0.000	0.000	0.000	0.000	0.006	-	-
Ampicillin (J01CA01)	Beta-lactam antibacterials, penicillins (J01C)	0.599	0.477	0.442	0.198	0.327	0.235	0.262	0.287	-10.74% (-19.79; -0.67)	Significant decrease (p=0.044)
Amoxicillin (J01CA04)		1.028	1.052	1.260	1.001	0.691	0.630	1.010	1.204	-1.82% (-11.09; 8.41)	n.s. (p=0.350)

Benzylpenicillin (J01CE01)		0.057	0.037	0.030	0.022	0.020	0.028	0.018	0.029	-9.77% (-19.22; 0.79)	Significant decrease (p=0.031)
Benzathine Benzylpenicillin (J01CE08)		0.000	0.015	0.000	0.000	0.000	0.000	0.000	0.000	-	-
Ampicillin and beta-lactamase inhibitor (J01CR01)		0.000	0.000	0.000	0.000	0.000	0.000	0.003	0.000	-	-
Amoxicillin and beta-lactamase inhibitor (J01CR02)		0.495	0.447	0.375	0.339	0.489	0.666	0.826	1.130	13.91% (2.61; 26.45)	Significant increase (p=0.043)
Sultamicillin (J01CR04)		0.000	0.000	0.000	0.000	0.000	0.000	0.000	0.000	-	-
Cefalexin (J01DB01)	Other beta-lactam antibacterials (J01D)	0.004	0.002	0.002	0.001	0.000	0.000	0.000	0.000	-	-
Cefazolin (J01DB04)		0.589	0.585	0.432	0.510	0.392	0.401	0.356	0.680	-2.32% (-10.93; 7.11)	n.s. (p=0.138)
Sulfaisodimidine (J01EB01)	Sulfonamides and trimethoprim (J01E)	0.066	0.175	0.180	0.199	0.200	0.120	0.000	0.000	-	-
Sulfamethoxazole and trimethoprim (J01EE01)		0.381	0.405	0.300	0.314	0.271	0.263	0.252	0.362	-3.83% (-9.60; 2.31)	Significant decrease (p=0.049)
Clindamycin (J01FF01)		0.003	0.003	0.002	0.003	0.005	0.004	0.004	0.005	8.94% (0.77; 17.77)	n.s. (p=0.071)
Gentamicin (J01GB03)		0.404	0.368	0.269	0.237	0.027	0.054	0.080	0.004	-42.29% (-58.36;-20.02)	Significant decrease (p=0.001)
Amikacin (J01GB06)		0.011	0.012	0.010	0.010	0.012	0.012	0.011	0.041	12.14% (-3.93; 30.89)	n.s. (p=0.138)
Metronidazole (J01XD01)		0.161	0.197	0.179	0.200	0.142	0.160	0.191	0.310	4.60% (-3.93; 13.90)	n.s. (p=0.274)
Tinidazole (J01XD02)		0.000	0.000	0.000	0.000	0.000	0.000	0.000	0.000	-	-
Ornidazole (J01XD03)		0.002	0.002	0.001	0.000	0.001	0.001	0.001	0.001	-	-
Nitrofurantoin (J01XE01)		0.330	0.305	0.274	0.207	0.292	0.284	0.270	0.475	2.89% (-6.04; 12.67)	n.s. (p=0.360)
Furazidin (J01XE03)		0.283	0.274	0.237	0.231	0.166	0.182	0.201	0.331	-1.86% (-10.61; 7.74)	n.s. (p=0.199)
Spectinomycin (J01XX04)		0.008	0.006	0.006	0.001	0.000	0.000	0.000	0.000	-	

ΩATC5 - Anatomical Therapeutic Chemical classification. αDID – Defined Daily Doses per 1000 inhabitants per day. ⁰APC – Average Annual Percent Change. *CI – Confidence Interval. [a]Mann-Kendall-test. **n.s. – not significant

Table S3. Consumption of the WHO AWaRe Access group antibiotics in the hospital sector

Substance (ΩATC5 code)	Pharmacological subgroup (ATC3 code)	DIDα								APC⁰ (95% CI*)	Trend analysis [a]
		2017	2018	2019	2020	2021	2022	2023	2024		
Doxycycline (J01AA02)	Tetracyclines (J01A)	0.070	0.064	0.019	0.088	0.058	0.035	0.035	0.089	0.05% (-19.63; 24.30)	n.s.** (p=0.452)
Tetracycline (J01AA07)		0.001	0.003	0.001	0.000	0.000	0.000	0.000	0.000	-36.35% (-51.74; -16.05)	n.s. (p=0.231)
Chloramphenicol (J01BA01)	Amphenicols (J01B)	0.004	0.004	0.003	0.004	0.002	0.001	0.001	0.001	-22.57% (-32.16; -11.63)	Significant decrease (p=0.001)
Thiamphenicol (J01BA02)		0.000	0.000	0.000	0.000	0.000	0.000	0.000	0.000	-	-
Ampicillin (J01CA01)	Beta-lactam antibacterials, penicillins (J01C)	0.033	0.045	0.004	0.034	0.016	0.014	0.015	0.000	-35.06% (-62.04; 11.07)	Significant decrease (p=0.045)
Amoxicillin (J01CA04)		0.118	0.012	0.010	0.153	0.145	0.069	0.043	0.284	24.21% (-20.47; 94.00)	n.s. (p=0.274)
Benzylpenicillin (J01CE01)		0.035	0.037	0.001	0.002	0.002	0.018	0.010	0.000	-34.43% (-66.30; 27.57)	n.s. (p=0.138)
Benzathine Benzylpenicillin (J01CE08)		0.000	0.000	0.000	0.000	0.000	0.000	0.000	0.000	-	-
Ampicillin and beta-lactamase inhibitor (J01CR01)		0.000	0.000	0.000	0.000	0.000	0.000	0.000	0.000	-	-
Amoxicillin and beta-lactamase inhibitor (J01CR02)		0.192	0.100	0.033	0.085	0.132	0.070	0.010	0.268	-7.36% (-38.93; 40.52)	n.s. (p=0.360)
Sultamicillin (J01CR04)		0.000	0.000	0.000	0.000	0.000	0.000	0.000	0.000	-	-
Cefalexin (J01DB01)	Other beta-lactam antibacterials (J01D)	0.000	0.000	0.000	0.000	0.000	0.000	0.000	0.000	-	-
Cefazolin (J01DB04)		0.478	0.485	0.457	0.455	0.315	0.275	0.296	0.212	-11.27% (-15.23; -7.13)	Significant decrease (p=0.001)
Sulfaisodimidine (J01EB01)		0.000	0.001	0.001	0.001	0.001	0.000	0.000	0.000	-	-

Sulfamethoxazole and trimethoprim (J01EE01)		0.045	0.007	0.004	0.013	0.048	0.025	0.039	0.067	24.21% (-11.96; 75.26)	n.s. (p=0.089)
Clindamycin (J01FF01)		0.000	0.000	0.000	0.000	0.000	0.000	0.000	0.000	-	-
Gentamicin (J01GB03)		0.518	0.493	0.431	0.504	0.338	0.265	0.286	0.131	-15.55% (-22.69; -7.75)	Significant decrease (p=0.028)
Amikacin (J01GB06)		0.096	0.059	0.065	0.071	0.079	0.015	0.028	0.063	-12.19% (-29.38; 9.19)	n.s. (p=0.199)
Metronidazole (J01XD01)	Sulfonamides and trimethoprim (J01E)	0.175	0.187	0.181	0.205	0.153	0.139	0.152	0.314	2.40% (-7.40; 13.24)	n.s. (p=0.452)
Tinidazole (J01XD02)		0.000	0.000	0.000	0.000	0.000	0.000	0.000	0.000	-	-
Ornidazole (J01XD03)		0.000	0.000	0.000	0.000	0.000	0.000	0.000	0.000	-	-
Nitrofurantoin (J01XE01)		0.003	0.002	0.003	0.001	0.001	0.007	0.001	0.001	-5.75% (-27.47; 22.47)	n.s. (p=0.138)
Furazidin (J01XE03)		0.001	0.001	0.001	0.001	0.000	0.000	0.001	0.001	-5.20% (-28.38; 25.47)	n.s. (p=0.734)
Spectinomycin (J01XX04)		0.000	0.000	0.000	0.000	0.000	0.000	0.000	0.000	-	-

[Ω]ATC5 - Anatomical Therapeutic Chemical classification. [α]DID – Defined Daily Doses per 1000 inhabitants per day. [0]APC – Average Annual Percent Change. *CI – Confidence Interval. [a]Mann-Kendall-test. **n.s. – not significant.

Table S4. World Health Organization AWaRe "Access" group antibiotics that are not listed in the WHO Essential Medicines List and/or essential medicines list for children and are not available in Kazakhstan

#	Substance	ATC5* code	Pharmacological group	Registered in Kazakhstan	Marketed in Kazakhstan
1	Pivampicillin	J01CA02	Penicillins with extended spectrum	No	No
2	Bacampicillin	J01CA06			
3	Epicillin	J01CA07			
4	Pivmecillinam	J01CA08			
5	Mecillinam	J01CA11			
6	Metampicillin	J01CA14			
7	Talampicillin	J01CA15			
8	Hetacillin	J01CA18			
9	Propicillin	J01CE03	Beta-lactamase sensitive penicillins		
10	Azidocillin	J01CE04			

11	Penamecillin	J01CE06	
12	Clometocillin	J01CE07	
13	Benzathine-phenoxymethylpenicillin	J01CE10	
14	Sulbactam	J01CG01	Beta-lactamase inhibitors
15	Cefaloridine	J01DB02	First-generation cephalosporins
16	Cefalotin	J01DB03	
17	Cefazedone	J01DB06	
18	Cefatrizine	J01DB07	
19	Cefapirin	J01DB08	
20	Cefradine	J01DB09	
21	Cefacetrile	J01DB10	
22	Cefroxadine	J01DB11	
23	Ceftezole	J01DB12	
24	Brodimoprim	J01EA02	Trimethoprim and derivatives
25	Sulfamethizole	J01EB02	Short-acting sulfonamides
26	Sulfadimidine	J01EB03	
27	Sulfapyridine	J01EB04	
28	Sulfafurazole	J01EB05	
29	Sulfanilamide	J01EB06	
30	Sulfathiazole	J01EB07	
31	Sulfathiourea	J01EB08	
32	Sulfamethoxazole	J01EC01	Intermediate-acting sulfonamides
33	Sulfadiazine	J01EC02	
34	Sulfamoxole	J01EC03	
35	Sulfadimethoxine	J01ED01	Long-acting sulfonamides
36	Sulfalene	J01ED02	
37	Sulfametomidine	J01ED03	
38	Sulfametoxydiazine	J01ED04	
39	Sulfamethoxypyridazine	J01ED05	
40	Sulfaperin	J01ED06	
41	Sulfamerazine	J01ED07	
42	Sulfaphenazole	J01ED08	
43	Sulfamazone	J01ED09	
44	Sulfadiazine/trimethoprim	J01EE02	

45	Sulfametrole/trimethoprim	J01EE03	Sulfonamide-trimethoprim-combinations
46	Sulfamoxole/trimethoprim	J01EE04	
47	Sulfadimidine/trimethoprim	J01EE05	
48	Sulfadiazine/tetroxoprim	J01EE06	
49	Sulfamerazine/trimethoprim	J01EE07	
50	Nifurtoinol	J01XE02	Nitrofuran and derivates

ATC5* – Anatomical Therapeutic Chemical classification, level 5

Table S5. Comparison of national standards of care for common infections at the primary care level with WHO AWaRe Antibiotic Book recommendations.

	**Kazakhstani standards of care**				**WHO AWaRe antibiotic book**			
**Disease**	**Indications**	**Antibiotic**	**Dose & frequency**	**Duration**	**Indications**	**Antibiotic**	**Dose & frequency**	**Duration**
**Acute sinusitis**	For bacterial infection	Ampicillin	250 or 500 mg, 4 times per day	Not indicated	Antibiotics are not required in most cases. They are indicated only in the presence of fever ≥39.0 °C, purulent nasal discharge, or facial pain lasting at least 3–4 consecutive days, in patients with "red flag" signs, or in those at increased risk of complications.	Amoxicillin	1 g, q8h	5 days
	For bacterial infection, mild to moderate cases	Amoxicillin + clavulanic acid	250mg+125mg q8h or 500mg+125mg q12h	Not indicated				
	For bacterial infection, mild to moderate cases	Amoxicillin + clavulanic acid	500 mg+125 mg q8h or 875 mg+125 mg q12h	Not indicated				
	For bacterial infection	Azithromycin	500mg, 1 time per day	3 days		Amoxicillin + clavulanic acid	500 mg+125 mg, q8h	5 days
**Community-acquired pneumonia**	For patients with mild disease and no comorbidities	Amoxicillin	500 mg, 3 times per day or 1 g, 2 times per day	5-10 days	In mild to moderate cases, first choice	Amoxicillin	1 g, q8h	5 days
	Second choice	Azithromycin	500 mg, 1 time per day	5-10 days		Phenoxymethy lpenicillin	500 mg, q6h	5 days
				5-10 days	In mild to moderate cases, second choice	Amoxicillin + clavulanic acid	875 mg+125 mg, q8h	5 days, consider longer treatment and look for complications such
						Doxycycline	100 mg, q12h	
		Clarithromycin	500 mg, 2 times per day		In severe cases, first choice	Cefotaxime	2 g, q8h, IV/IM	

						Ceftriaxone	2 g, q24h, IV or 1g, q24h, IM	as empyema, if patient not clinically stable at day 5
					In severe cases, second choice	Amoxicillin + clavulanic acid	1 g+200 mg, q8h, IV or q6h	
						Clarithromycin	500 mg, q12h, oral or IV	
**Chronic bronchitis/ chronic obstructive pulmonary disease**	Antibiotics are prescribed in the presence of a bacterial infection, as evidenced by an increase in sputum volume, sputum purulence, the presence of systemic symptoms (e.g., fever, chills, weakness, decreased appetite, reduced performance), and elevated laboratory markers of systemic inflammation (e.g., leukocytosis with a neutrophilic shift, CRP[Ω])	Amoxicillin + clavulanic acid	500 mg+125 mg, q8h or 875 mg+125 mg, q12h	5-7 days	Antibiotics are considered in patients with dyspnea and increased volumes of purulent sputum.<br>In mild to moderate cases:	Amoxicillin	500 mg, q8h	5 days
		Cefuroxime	250-500 mg, 2 times per day	5-7 days		Cefalexin	500 mg, q8h	5 days
		Cefixime	400 mg, once per day or 200 mg, 2 times per day	5-7 days		Doxycycline	100 mg, q12h	5 days
		Azythromycin	500 mg, once per days	3 days	In severe cases			
		Ceftriaxone	1 g, once per day, IM**	5-7 days		Amoxicillin + clavulanic acid	500 mg+125 mg, q8h	5 days
**Otitis media**	Antibiotics are prescribed if fever and severe pain is present	Ampicillin	250 or 500 mg, 4 times per day	N/A	Antibiotics are considered in the presence of severe symptoms (e.g., systemic illness, ear pain despite analgesics, or fever ≥39.0 °C).	Amoxicillin	500 mg, q8h	5 days
		Amoxicillin + clavulanic acid	250mg+125mg q8h or 500mg+125mg g12h	N/A				
		Azithromycin	500mg, 1 time per day	3 days		Amoxicillin + clavulanic acid	500 mg+125 mg, q8h	5 days
**Pharyngitis**	Systemic antibiotic therapy is used to prevent purulent complications, descending infection in cases of severe	Not indicated	Not indicated	Not indicated	Antibiotics are not required in most cases.The only clear indication for antibiotic treatment is to reduce the probability of	Amoxicillin	500 mg, q8h	3 days if adequate source control is achieved; otherwise, 5 days.
						Phenoxymethy lpenicillin	500 mg, q6h	
						Cefalexin	500 mg, q8h	
						Clarithromycin	500 mg, q12h	

	general reaction with fever				developing rheumatic fever in endemic settings.			
**Ulcerative gingivitis**	With symptoms of intoxication of moderate and severe cases in treating ulcerative gingivitis.	Tinidazole	500 mg, 2 times per day	5 days		Amoxicillin	500 mg, q8h	
						Phenoxymethylpenicillin	500 mg, q6h	
**Periodontitis**	Not indicated	Doxycycline	200 mg, 2 times per day for first 2 days, then 100 mg, 2 times per day	10 days	Antibiotics are prescribed for severe cases that include systemic signs of infection (fever ≥ 38.0 °C, tachycardia, facial swelling, inability to open the mouth).	Amoxicillin	500 mg, q8h	3 days if adequate source control is achieved; otherwise, 5 days.
		Tinidazole	500 mg, 2 times per day	5 days		Phenoxymethylpenicillin	500 mg, q6h	
**Lower urinary tract infections: cystitis and urethritis**	Acute cystitis of mild to moderate severity without complications		0.5 per os, once	7-10 days		Amoxicillin+clavulanic acid	500 mg+125 mg, q8h	3-5 days
		Levofloxacin	0.75 per os, once	5 days		Nitrofurantoin	100 mg, q12h (modified release formulation); 50 mg, q6h (immediate release formulation)	5 days
			0.5-0.75 per os, 2 times	7-10 days		Sulfamethoxazole+trimethoprim	800 mg+160 mg, q12h	3 days
		Ciprofloxacin	1.0*1 per os, once	5 days				
	Only with known sensitivity of the pathogen	Amoxicillin + clavulanic acid	0.5-0.125 per os, 3 times	14 days	Antibiotic treatment is recommended if compatible clinical presentation AND a positive test (positive urine leucocytes/leucocyte esterase or positive urine culture) are present.			
		Cefixime	0.4 per os, once	7-10 days				
	Acute bacterial cystitis without complications	Fosfomycin	3.0 g per os	NA				
		Furazidin	0.1 per os, 3 times	5 days				
		Nitrofurantoin	0.1 per os, 3, times	5 days				
		Ofloxacin	0.2 per os, 2 times	3 days		Trimethoprim	200 mg, q12h	3 days

		Ciprofloxacin	0.5 per os, 2 times	3 days				
		Levofloxacin	0.5 per os, once	3 days				
		Cefixime	0.4 per os, once	5 days				
**Impetigo**	Antibiotics are prescribed for extensive or recurrent impetigo, in cases of failure of topical therapy, or in the presence of systemic symptoms (e.g., fever, malaise) or regional complications (e.g., lymphadenitis, lymphangitis).	Amoxicillin	500-750 mg, 2 times per day, orally	7-10 days	Prescription for mild bacterial impetigo, erysipelas, cellulitis	Amoxicillin+ clavulanic acid	500 mg+125 mg, q8h	5 days; longer durations may be required if there is no clinical improvement or if an underlying medical condition is present
		Cefazolin	1000 mg in 2-4 takes, IM	7-10 days		Cefalexin	500 mg, q8h	
		Ceftriaxone	1000-2000 mg, once per day, IM	7-10 days				
		Erythromycin	1000-4000mg in 4 takes, orally	5-14 days	In cases of localized non-bullous impetigo, topical treatment is preferred over oral antibiotics whenever possible.	Cloxacillin	500 mg, q6h	
		Azithromycin	1 g for 1st day, then 500 mg for 2-5th day	5 days				
		Clarithromycin	500-1000 mg in 2 takes, orally	7-10 days				
		Doxycycline	100 mg, orally	10-14 days				
		Ciprofloxacin	250-500 mg, 2 times per day, orally	5-15 days				
		Levofloxacin	250-500 mg, 1-2 times per day, orally	7-14 days				
		Ofloxacin	200-400 mg, 2 times per day, orally	7-10 days				
		Gentamicin	3-5mg/kg/day, 2-4 times per day, IM	7-10 days				
**Skin abscess, boil and carbuncle**	Indications for antibiotic therapy are not specified.	Amoxicillin + clavulanic acid	500 mg+125 mg, 3 times per day	Not indicated	Prescription for mild bacterial impetigo, erysipelas, cellulitis, where the treatment of skin abscess is described	Amoxicillin+ clavulanic acid	500 mg+125 mg, q8h	5 days; longer durations may be required if there is no clinical improvement or if an underlying medical condition is present.
		Cephalexin	500 mg, 4 times per day			Cefalexin	500 mg, q8h	
		Cefuroxim	750 mg; 500 mg, 2 times per days			Cloxacillin	500 mg, q6h	
		Erythromycin	500mg, 4 times per day					
		Oxacillin	500 mg, 4-6 times per day, IV°/IM					
		Cefazolin	1000 mg, 3-4 times per day, IV/IM					
		Ceftriaxone	1000 mg, once per day, IM					

*N/A – non-applicable. ΩCRP – C-reactive protein. **IM – intramuscular. °IV – intravenous.

Table S6. STROBE checklist.

Section	Item No	Recommendation	Location in manuscript
Title and abstract	1	(*a*) Indicate the study's design with a commonly used term in the title or the abstract	Page 1, Title
		(*b*) Provide in the abstract an informative and balanced summary of what was done and what was found	Page 1, Abstract
Introduction			
Background/rationale	2	Explain the scientific background and rationale for the investigation being reported	Page 2, Introduction
Objectives	3	State specific objectives, including any prespecified hypotheses	Page 3, Introduction
Methods			
Study design	4	Present key elements of study design early in the paper	Page 11, Subsection 4.1
Setting	5	Describe the setting, locations, and relevant dates, including periods of recruitment, exposure, follow-up, and data collection	Page 11-12, Subsections 4.2 and 4.3
Participants	6	(*a*) Give the eligibility criteria, and the sources and methods of selection of participants	Not applicable
Variables	7	Clearly define all outcomes, exposures, predictors, potential confounders, and effect modifiers. Give diagnostic criteria, if applicable	
Data sources/ measurement	8*	For each variable of interest, give sources of data and details of methods of assessment (measurement). Describe comparability of assessment methods if there is more than one group	Page 12. Subsection 4.4
Bias	9	Describe any efforts to address potential sources of bias	Not applicable
Study size	10	Explain how the study size was arrived at	
Quantitative variables	11	Explain how quantitative variables were handled in the analyses. If applicable, describe which groupings were chosen and why	Page 12. Subsection 4.4
Statistical methods	12	(*a*) Describe all statistical methods, including those used to control for confounding	
		(*b*) Describe any methods used to examine subgroups and interactions	Not applicable
		(*c*) Explain how missing data were addressed	
		(*d*) If applicable, describe analytical methods taking account of sampling strategy	

		(*e*) Describe any sensitivity analyses	
Results			
Participants	13*	(a) Report numbers of individuals at each stage of study—eg numbers potentially eligible, examined for eligibility, confirmed eligible, included in the study, completing follow-up, and analysed	Not applicable
		(b) Give reasons for non-participation at each stage	
		(c) Consider use of a flow diagram	
Descriptive data	14*	(a) Give characteristics of study participants (eg demographic, clinical, social) and information on exposures and potential confounders	
		(b) Indicate number of participants with missing data for each variable of interest	
Outcome data	15*	Report numbers of outcome events or summary measures	
Main results	16	(*a*) Give unadjusted estimates and, if applicable, confounder-adjusted estimates and their precision (eg, 95% confidence interval). Make clear which confounders were adjusted for and why they were included	Pages 3,4. Subsection 2.1
		(*b*) Report category boundaries when continuous variables were categorized	Not applicable
		(*c*) If relevant, consider translating estimates of relative risk into absolute risk for a meaningful time period	
Other analyses	17	Report other analyses done—eg analyses of subgroups and interactions, and sensitivity analyses	
Discussion			
Key results	18	Summarise key results with reference to study objectives	Pages 9-10, Discussion
Limitations	19	Discuss limitations of the study, taking into account sources of potential bias or imprecision. Discuss both direction and magnitude of any potential bias	Page 11, Discussion
Interpretation	20	Give a cautious overall interpretation of results considering objectives, limitations, multiplicity of analyses, results from similar studies, and other relevant evidence	Pages 9-10, Discussion
Generalisability	21	Discuss the generalisability (external validity) of the study results	
Other information			
Funding	22	Give the source of funding and the role of the funders for the present study and, if applicable, for the original study on which the present article is based	Page 14, Funding section
